# Cause of Yellow Fever

**Published:** 1877-04

**Authors:** 


### Editorial.

### CAUSE OF YELLOW FEVER.

In order to the proper understanding of medical, as well as other discussions, a knowledge of the terms used is indispensable—a uniform definition must be adopted. It is said that yellow fever was imported to Savannah. By this are we to understand that cases of the disease were brought to that city from some other port, or that the poisonous material was brought, and in sufficient quantity to infect the whole people as an epidemic? In the report of our State Board of Health, the term seems to be used ambiguously. The context, in one instance, would seem to favor the former definition, while in another the latter appears to be intended. For example, the fever in Brunswick, as stated in the report, "first appeared in connection with the vessels in the harbor, and gradually spread throughout the town from that quarter." This spreading "throughout the town," is mentioned, doubtless, to prove the communication from one person to another. The case of Lufburrow, who contracted the disease at Station 4½ Central Railroad, by sleeping in a car brought from Savannah, is intended to show that the malaria or poison may be transported. But it is said "the disease did not spread." By this we understand that the disease was not communicable, from one person to another.

Here then we have two instances of the onset of the fever.

In one case the disease was said to be imported *and spread*, which it could not have done under the circumstances, except by personal communication or the local malaria. In the Lufburrow case the poison, not the disease, was said to be imported, and *did not spread*; that is, of course, it was not communicated to others, and there was no local malaria in the neighborhood. If it is personally communicable at one time and place, why is it not at another? Of course persons are more susceptible to diseases under certain circumstances, but when we find in hundreds and even thousands of instances, contact without communication, and not one instance reported in which the disease spread in localities where no malaria existed, we must conclude that malaria is indispensable to the *spreading*. Moreover, malaria was reported and known to exist in the immediate range of the first cases that occurred in Savannah and Macon, and may with propriety be set down as the cause of all the cases that occurred.

Other important facts bearing on this subject must be kept in view, in order to arrive at proper clonclusions. It will be remembered that not only in the epidemic of 1876, but whenever yellow fever has existed in this State, the various other forms of malarial fever were found in connection with it. Furthermore, in the epidemics of 1854 and 1876, the ordinary conditions for the production of malaria were notoriously present in Savannah and Augusta, such as the overflow of the Savannah river and other streams by which marshes, lagoons, etc., were filled, and the water in them gradually subsided under a very high temperature.

The opinion of the Board of Health on this subject strikes us as decidedly equivocal, as before intimated. While there is a *quasi* confession of local malarial origin in one part of the report, in another it is virtually denied in the most emphatic language. Says the report, "and the evidence that the disease may have originated from, or was aggravated by, local causes, is sufficiently strong to arrest our most intelligent attention." Again, the Board thinks there was "no reason why this or any other disease possible to the locality might not have originated in Savannah from the great neglect of its internal hygiene." On the other hand, the Board says: "The question is constantly asked whether any system of quarantine is likely to be effective in preventing invasions of yellow fever. This question may be confidently answered in the affirmative, and it may be stated that the objections to quarantine are largely based upon ignorance of the facts connected with its operation or of the origin of diseases it is designed to limit," etc.

Now if local malaria causes yellow fever, as conceded above, and also in the case of Mr. Lufburrow by malaria brought from Savannah in a car, how is it possible to prevent the disease by quarantine? We confess to "ignorance of the facts connected with its operation," if quarantine will prevent the formation of malaria under circumstances which surrounded Savannah, Augusta, and Macon last summer. The quarantine, it strikes us, would be more reasonably directed against the interior, so as to prevent the rushing water from overflowing and standing upon the marshes and pools by which this pestiferous material is formed.

Malaria generated in Georgia does sometimes produce yellow fever, or it does not. This is the question to be settled. If it does, quarantine cannot prevent the disease, and our labors would be more profitably directed toward the prevention of the condition likely to produce the poison.

For the quarantinists, the report of the Board is unfortunate in alluding to the report of the Health Officer of New York for 1876 as proof of the prevention of yellow fever by quarantine. If importation of cases of the disease only was necessary to infect the city, New York would certainly have been scourged by an epidemic. Three hundred and fifty-five cases were imported to New York harbor, besides two cases by rail introduced into the heart of the city; and yet no well authenticated case occurred among the residents. The Board, also, in a note giving an abstract of the Health Officer's report, misstates the facts—unintentionally no doubt—in regard to the limitation of the vessels to cargo and crew. Passengers were not prohibited except on vessels from Brunswick, Savannah, and Charleston, which would make the trip to New York in less time than the usual period of incubation. Even this precaution did not seem to be necessary, as the two cases above alluded to going through by rail certainly gave every chance for communication, if such were likely to occur, and yet without detriment to the city.

It appears from a tabulated statement of the Board as found in the report, that the idea of importation of yellow fever existed for nearly two hundred years! The first account given of the disease is that of an epidemic in Boston in the year 1693, which was said to have resulted from importation of a case from Barbadoes. Authority for this opinion of importation may therefore be traced back nearly two centuries, and continuously down to the report of our State Board of Health for 1876. In connection with these authorities, we remember the biblical advice, "ask for the old paths—walk therein," and yet we have an ungovernable propensity to inquire as to the correctness of the opinion and the direction of the paths. Still more ancient was the opinion that the touching of a wound with the weapon that made it would prove salutary. The same tendency has continued to the present day, and now attacks our credulity in the form of a buckeye in the pocket for the cure of hemorrhoids.

The truth is, this idea of importation is plausible. Commercial intercourse is very general; nay, we may say universal, directly or indirectly. It is hard to get off the line of commercial intercommunication, and it matters not where the fever may appear, the transfer of goods from, or personal communication with, some tropical port where fever exists the year round, may be traced. A ship was in port of Charleston from Havana, and an epidemic of yellow fever followed—the sword was slightly passed over the wound it had made, and the healing process followed. Now, in these instances we forget the fact, that often ships from infected ports, with yellow fever aboard, land at Atlantic ports in hot weather, as at New York last summer, and *no epidemic* follows; the sword is not applied to the wound, and yet it heals; the buckeye is worn in the pocket for years, and yet the piles continue.

The cities of our Atlantic coast affected with the epidemic in 1876, had no unusual intercourse with tropical yellow fever ports, and perhaps less than previous summers, when no disease followed. The unusual amount of rain during the summer, and excessively warm weather, satisfactorily account for the scourge.

We find our views on this subject corroborated, in the main, by those of Surgeon Robert T. Murray, as expressed in his report of the epidemic of yellow fever at Key West in 1875, to the Supervising Surgeon-General at Washington. The importation doctrine is virtually condemned by Dr. Murray, and conclusive evidence of its indigenous origin afforded. The treatment he adopted is that which is universaily used for the cure of other forms of malarial fever—quinine, five to ten grains every four hours, being one of the principal remedies. The death-rate was about twenty per cent., showing less than the usual mortality in epidemics of malignant fever.

The report closes with the remark, "that it is absolutely non-contagious and non-infectious in the sense that small-pox is contagious and typhus is infectious. Also that when the disease occurs, sick and well should be removed to healthy localities without delay."

Brunswick is said to afford incontestible evidence of importation, not only in the case of Mrs. West who was taken sick the day after going aboard the "Ed Johnson," but in the sickness and death of Capt. Bean, and in the first cases occurring in the neighborhood where ballast from the "Spanish vessel" "Maretta" was discharged, and spreading eastward against the prevailing wind.

On a former occasion we called in question the possibility of one day being sufficient time of incubation, and upon further investigation we find that no fever had been on the "Johnson," nor even the Spanish vessel "Maretta," since her arrival nor on her voyage, and furthermore that Capt. Bean, of the former vessel, who died of the fever, had come to Brunswick ten or fifteen days before his attack, from the North, by railroad, passing through Atlanta; and that his vessel, in command of the mate, was taking her cargo when he arrived. He was taken sick at his lodgings in town, and was carried to the hotel where he died.

We also learn from a reliable physician of Brunswick that serious cases of fever occurred before the "Johnson" or "Maretta" came in port, on account of which he had sent his family off about the first week of August.

He also states that brake-bone fever prevailed a few years ago, under the prevalence also of an east wind, as that of last year. This could not have been imported, and why the fever by or before the first of August in 1876, and the brake-bone fever previously, if not the result of malaria? This poison, then, is produced on the coast, and is wafted from the rice fields at a distance or from some other point of its production. This is sufficient to account for yellow-fever, brakebone, malignant remittent, intermittent or hæmaturial fever.

